# Virtual Screening of Peptide and Peptidomimetic Fragments Targeted to Inhibit Bacterial Dithiol Oxidase DsbA

**DOI:** 10.1371/journal.pone.0133805

**Published:** 2015-07-30

**Authors:** Wilko Duprez, Prabhakar Bachu, Martin J. Stoermer, Stephanie Tay, Róisín M. McMahon, David P. Fairlie, Jennifer L. Martin

**Affiliations:** Division of Chemistry and Structural Biology, Institute for Molecular Bioscience, University of Queensland, Brisbane, Queensland, Australia; University of Minnesota, UNITED STATES

## Abstract

Antibacterial drugs with novel scaffolds and new mechanisms of action are desperately needed to address the growing problem of antibiotic resistance. The periplasmic oxidative folding system in Gram-negative bacteria represents a possible target for anti-virulence antibacterials. By targeting virulence rather than viability, development of resistance and side effects (through killing host native microbiota) might be minimized. Here, we undertook the design of peptidomimetic inhibitors targeting the interaction between the two key enzymes of oxidative folding, DsbA and DsbB, with the ultimate goal of preventing virulence factor assembly. Structures of DsbB - or peptides - complexed with DsbA revealed key interactions with the DsbA active site cysteine, and with a hydrophobic groove adjacent to the active site. The present work aimed to discover peptidomimetics that target the hydrophobic groove to generate non-covalent DsbA inhibitors. The previously reported structure of a *Proteus mirabilis* DsbA active site cysteine mutant, in a non-covalent complex with the heptapeptide PWATCDS, was used as an *in silico* template for virtual screening of a peptidomimetic fragment library. The highest scoring fragment compound and nine derivatives were synthesized and evaluated for DsbA binding and inhibition. These experiments discovered peptidomimetic fragments with inhibitory activity at millimolar concentrations. Although only weakly potent relative to larger covalent peptide inhibitors that interact through the active site cysteine, these fragments offer new opportunities as templates to build non-covalent inhibitors. The results suggest that non-covalent peptidomimetics may need to interact with sites beyond the hydrophobic groove in order to produce potent DsbA inhibitors.

## Introduction

The recent emergence of ‘extremely drug resistant’ bacterial pathogen strains is a major public health concern [1] exacerbated by the low number of newly approved drugs to treat bacterial infections [2–4]. The World Health Organization has warned that we are entering a “post-antibiotic era” where minor infections will be deadly [5], while US President Obama issued an Executive Order to combat antimicrobial resistance [6]. Since the early 1980s newly approved antibiotics, with the exception of six classes, have been analogues of previously released scaffolds [7]. Moreover the six new classes [8–13] target Gram-positive pathogenic bacteria, contributing to an urgent need to develop new treatments aimed at infectious Gram-negative bacteria, particularly those among the “ESKAPE” pathogens [1]. Due to a shortage of novel naturally-occurring antibiotics, efforts have been made to design new antimicrobial scaffolds with different modes of action [14–18]. One approach is to target bacterial virulence pathways, which are thought to be less likely to induce resistance mechanisms [19]; neutralizing pathogenicity without impeding bacterial viability might limit adaptive resistance to the drug [20] and also reduce the impact on host native microbiota [21].

**Fig 1:**
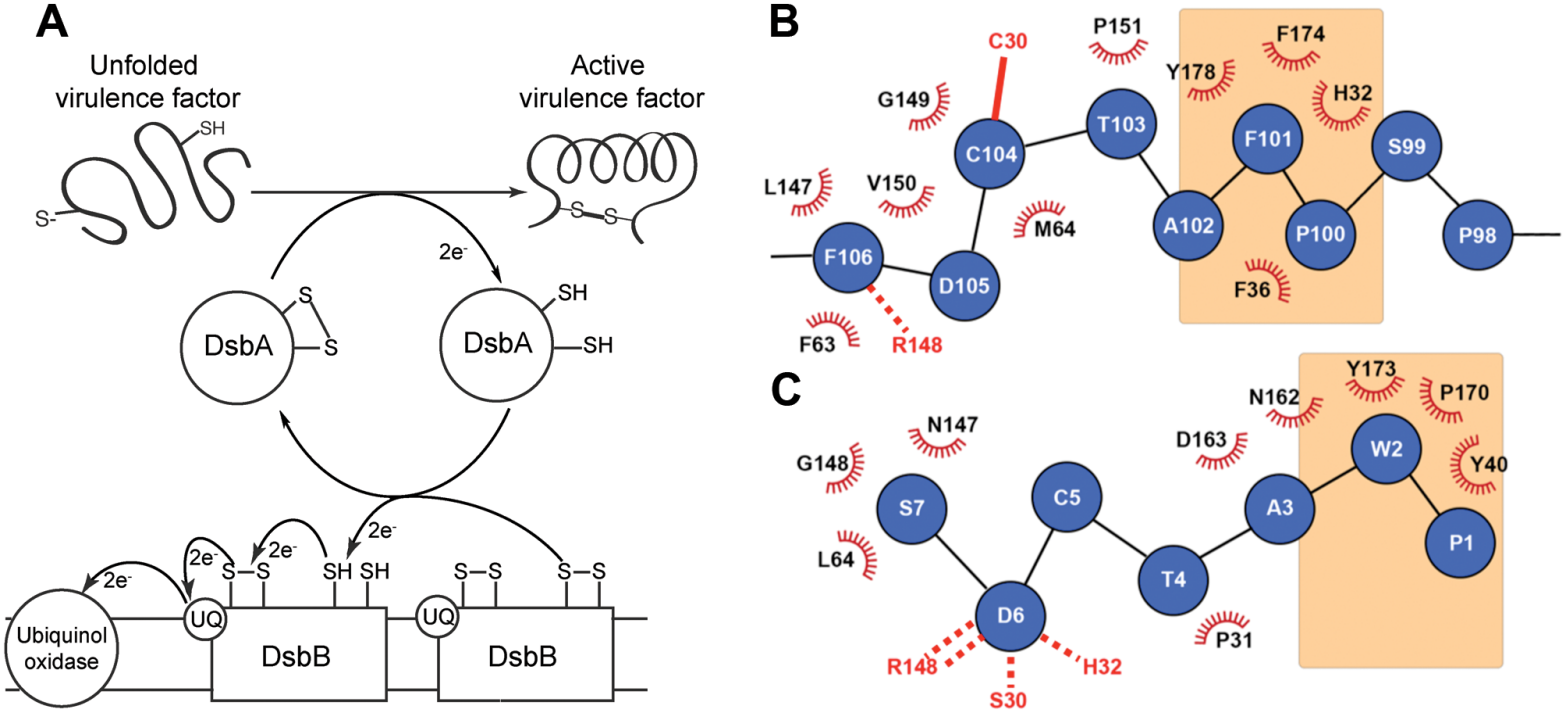
The DsbA-DsbB interaction. **A.** Schematic showing the proposed mechanism of oxidative folding in the periplasm of Gram-negative bacteria. DsbA catalyses the formation of a disulfide bond in a protein substrate, then interacts with DsbB to which it transfers electrons so that DsbA is regenerated into its active oxidized state. The electrons are subsequently transferred from DsbB to ubiquinone (UQ) and ultimately to the respiratory complex. **B.** The binding interface between EcDsbA (black and red) and EcDsbB loop P2 (blue) derived from the crystal structure of the EcDsbAC33A:EcDsbBC130S complex [37]. The EcDsbA hydrophobic groove residues are highlighted in orange shading, the intermolecular disulfide bond is shown as a solid red line and the hydrogen bond with the *cis*Pro loop is shown as a dashed red line. **C.** The binding interface between PmDsbA (black and red) and the peptide PWATCDS (blue) from the crystal structure of the complex [38]. In this complex there is no disulfide bond as the active site cysteine of PmDsbA was mutated to Ser (S30). The peptide Cys5 residue points away from the binding interface. Residues W2 and P1 of the peptide both interact with the hydrophobic groove (in orange) and these interactions were used as the target for this peptidomimetic design.

The periplasmic oxidative folding machinery that catalyses protein folding through disulfide bond formation is a potential target for antivirulence therapeutics and is widespread in Gram-negative bacteria [22]. Substrates of the pathway include components essential for pili formation and motility, host cell adhesion, toxin production, and secretion [23]. The archetypal machinery characterized in *Escherichia coli* K12 (Fig 1A) involves Dsb (disulfide bond forming) proteins [24, 25]. DsbA is a dithiol oxidase comprising a thioredoxin (TRX) domain and an inserted α-helical domain [26, 27]; a C*XX*C motif (^30^CPHC^33^ in *E*. *coli*) forms the catalytic site [28] together with a *cis*Pro loop and a groove formed from hydrophobic residues including Phe36, Phe174 and Tyr178. In the active oxidized state, *E*. *coli* DsbA (EcDsbA) has a disulfide bond between Cys30 and Cys33 which is transferred to a substrate through bimolecular nucleophilic transfer (SN2) (Fig 1A) [29–31]. Oxidative folding of the substrate converts EcDsbA to the inactive reduced form, which can then interact with the periplasmic loop P2 of transmembrane partner EcDsbB (Fig 1A) [32]. The EcDsbA-EcDsbB interaction regenerates the oxidized state of EcDsbA through SN2 transfer of electrons to EcDsbB [33, 34]. Inhibition of the EcDsbA-EcDsbB interaction would block oxidation of EcDsbA and thereby block oxidative folding of virulence factors. Accordingly, the phenotype of *d*s*bA/dsbB* null uropathogenic *E*. *coli* (UPEC) cells is severe attenuation of virulence in a mouse infection model, though bacteria remain viable [35]. Similarly, mice infected with a *d*s*bA* mutant of *B*. *pseudomallei* all survived whereas mice infected with wildtype all died [36].

The crystal structure of the EcDsbA-EcDsbB complex has been determined, through use of a covalent complex that trapped the otherwise transient interaction between the two proteins [39–41]. The structure revealed details of the intermolecular disulfide bond between EcDsbA Cys30 and EcDsbB Cys104, as well as a hydrogen bond between main chain atoms of EcDsbA Arg148 (on the *cis*Pro loop) and EcDsbB Phe106, and hydrophobic contacts between EcDsbB Pro100 and Phe101 and the EcDsbA hydrophobic groove (Fig 1B). We have identified peptides that bind to oxidized EcDsbA with low micromolar affinity (*K*d values 2–20 μM), but they all required a cysteine for inhibition of EcDsbA, suggesting that they targeted an active site cysteine in order to inhibit the enzyme [42]. We have also co-crystallized the peptide PWATCDS with a Cys30Ser mutant of *Proteus mirabilis* DsbA to generate a non-covalent complex (no disulfide bond is formed) (Fig 1C) [38]. In this non-covalent complex, the Pro1 and Trp2 residues of PWATCDS interact with the hydrophobic groove in a manner similar to that described for the EcDsbB periplasmic loop P2 interaction with EcDsbA (Fig 1C).

In the present work, we explored the importance of the DsbA hydrophobic groove for inhibition by designing and developing small peptide-derived molecules predicted by computer modeling to bind to this region. EcDsbA and PmDsbA are very similar proteins [38] so we used the high-resolution PmDsbAC30S-PWATCDS non-covalent protein-heptapeptide complex structure as the starting point for an *in silico* virtual screen of a peptidomimetic library. Our goal was to move from peptides to more ‘drug-like’ compounds, by designing and screening peptidomimetics. The resulting *in silico* hit and nine derivatives were synthesized and their affinities and inhibitor potencies for native EcDsbA were measured using a combination of differential scanning fluorimetry, isothermal titration calorimetry (ITC) and an enzyme assay. The compounds were weak inhibitors of EcDsbA, suggesting that additional binding interactions will be required to generate significant inhibitor potency.

## Materials and Methods

### Virtual Docking

The 1.6 Å resolution crystal structure of the PmDsbAC30S-PWATCDS protein-heptapeptide complex (PDB code 4OD7 [38]) was used to dock putative ligands in the substrate-binding active site of DsbA. The bound peptide was removed from the structure and hydrogen atoms were added using the Hermes interface in GoldSuite 5.1 [43]. Additionally the indole ring system of Trp2 of the bound peptide was separately stored in the same XYZ coordinate space to serve as a template file for the template docking mode within GOLD. The PWA tripeptide was replaced with a small targeted library of 10 compounds based on both D- and L-tryptophan cores, and both hydrophobic and hydrophilic C- and N-terminal capping groups. These candidate virtual ligands were prepared from 2D ChemDraw (CS ChemBioDraw Ultra 12.0. Cambridge Scientific Computing) representations via SMILES strings, and minimum energy conformers were prepared using OMEGA2 v2.4.6 [44–46] and the mmff94s forcefield. Docking with GOLD 5.1 was performed using the standard precision settings and the default CHEMPLP scoring function. Docking results were visualized in Pymol [47–49]. The initial template docking with the indole fragment in its previously determined location [38] was carried out with the template constraint set to the default values and progressively reduced to allow for better fit docking and to not overly bias in favour of the observed crystal structure bound conformation of the Trp. The backbone amine and carbonyl of Trp2 in the bound full length peptide do not form hydrogen bonds with PmDsbAC30S [38], allowing replacement and removal to explore whether simpler small molecules could bind with conformations differing from the full length peptide, with only the indole ring of Trp2 used as a hydrophobic anchor. The steady reduction in the template constraint also allowed subtle changes to arise that may provide additional interactions in the case of smaller molecules, which would reasonably be assumed to adopt multiple possible binding conformations. Indeed if the constraint was completely reduced, other conformations were observed (up to 4 out of 10 docking poses), some of which included extra H-bonds with the protein, particularly with the charged molecules. Nevertheless, the dominant interactions in this portion (PWA) of the heptapeptide (PWATCDS) were the hydrophobic interactions of the Pro and Trp side chain fragments.

### Peptidomimetic synthesis

**Fig 2:**
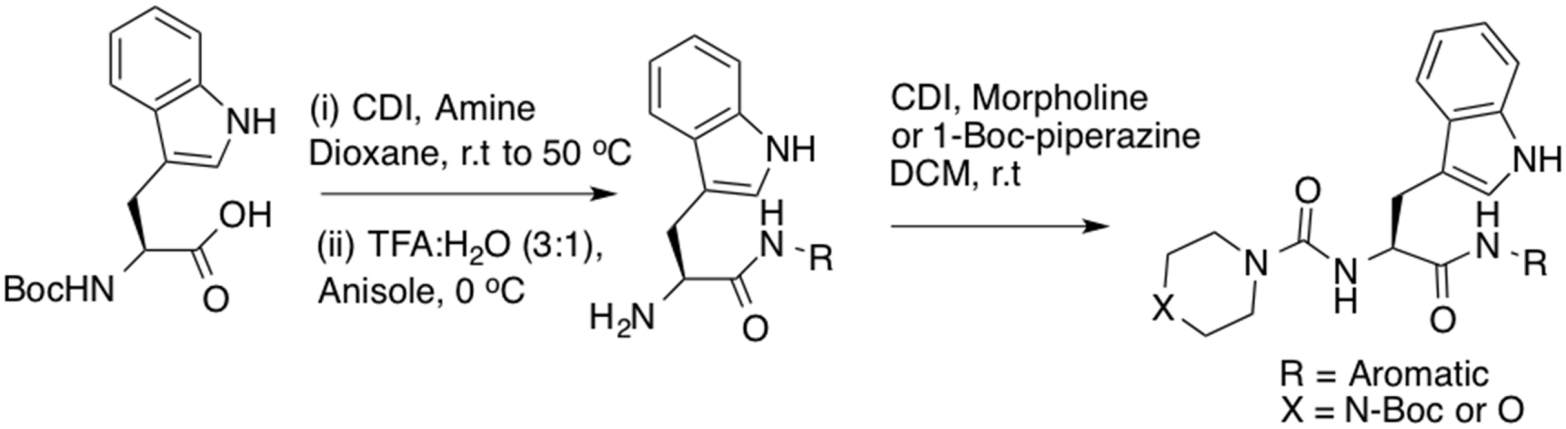
Synthetic route for the tripeptide peptidomimetics.

All purchased chemicals were used without further purification. All solvents were HPLC grade. Boc-Trp-OH (1.00 mmol) was dissolved in 10 mL of dioxane under nitrogen. Carbonyldiimidazole (1.02 mmol) was then added in a stepwise manner and the resulting mixture stirred for 3 h at room temperature and then heated to 50°C for 30 min. The desired amine (1.03 mmol) was added to the reaction mixture at room temperature and stirring was continued for 48 h (Fig 2). The solvent was removed under reduced pressure and crude product was extracted with ethyl acetate (3 x 20ml). The organic extracts were washed with 1M hydrochloric acid (15 mL), saturated sodium bicarbonate solution (15 mL) and water (15 mL), then dried over anhydrous MgSO_4_ and the solvent was removed under reduced pressure. To this crude compound in water (1 mL) was added anisole (2.00 mmol). The solution was then cooled to 0°C prior to addition of trifluoroacetic acid (30.00 mmol) in 1 mL of water. After stirring for 1 h at 0°C, the reaction mixture was allowed to warm to room temperature and stirring was continued for 12 h. The reaction mixture was then diluted with ethyl acetate (15 mL) and washed with saturated sodium bicarbonate solution (15 mL), water (15 mL) and brine solution (15 mL) and dried over anhydrous MgSO_4_. The solvent was removed under reduced pressure to afford the crude free amine which was directly used in the next step without further purification. To a solution of the above crude amine in dichloromethane (5 mL) was added carbonyldiimidazole (1.2 mmol) under nitrogen at room temperature. After 3 h stirring, morpholine or 1-Boc-piperazine (1.55 mmol) was added to the reaction mixture and stirred for another 12 h at room temperature. The solvent was removed under reduced pressure, diluted with ethyl acetate (15 mL) and washed with 1M hydrochloric acid (15 mL), saturated sodium bicarbonate solution (15 mL), brine solution (15 mL) and dried over anhydrous MgSO_4_. The crude product was then purified by preparative HPLC (Gradient 0 to 100% of 95/5 acetonitrile/water solution over 25 min) and freeze-dried. NMR spectral data of all synthesized compounds **1**–**10** are included in the supporting information (S1–S10 Figs). NMR spectra were recorded on Bruker Avance DRX-600 and Varian 400 MHz spectrometers at 298 K with TMS as internal standard. High-resolution mass spectrometry (HRMS) was performed on a Bruker micro-TOF by direct infusion in acetonitrile/H_2_O 70:30 at 3 μL/min using sodium formate clusters as an internal calibrant. Semi-preparative RP-HPLC purification of the compounds was performed using a Waters Delta 600 chromatography system fitted with a Waters 486 tuneable absorbance detector with detection at 214 nm. Purification was performed by eluting with solvents A (0.1% TFA in water) and B (9:1 CH_3_CN:H_2_O, 0.1% TFA) on a Vydac C18 250 x 22 mm (300 Å) steel jacketed column at 20 mL/min. NH peak of Indole is not observed in some of the compounds in CDCl_3_ due to peak broadness.

### Protein expression and purification

Wild type *E*. *coli* DsbA was expressed and purified in our laboratory as previously described [42, 50].

*E*. *coli* membrane preparations containing over-expressed EcDsbB (GenBank accession number AAC74269) were produced as previously reported [51], and resuspended in PBS buffer containing 10% glycerol.

### Differential Scanning Fluorimetry

All differential scanning fluorimetry experiments were performed on a ViiA 7 Real-Time PCR instrument (Life Technologies, Applied Biosystems Division) using 384 well MicroAmp Clear Optical Plates (Life Technologies, Invitrogen Division). Oxidised EcDsbA (25 mM HEPES pH 7.4, 50 mM NaCl) was prepared at a final concentration of 2.5 μM, in the presence of either 5% (v/v) DMSO (reference) or 2, 1, 0.5, 0.25, 0.125 or 0.0625 mM of the test compound (also in a final concentration of 5% DMSO (v/v.)) A 5000x stock of SYPRO Orange Protein Gel Stain (Life Technologies, Invitrogen Division) was diluted to a final concentration of 5x. The final reaction volume was 20 μL. For each condition, five technical replicates were included. Non-protein control reactions (DMSO or compound, and dye in the absence of EcDsbA) were also included to monitor compound-dye interactions. Plates were sealed with Axygen Ultra Clear Pressure Sensitive Sealing Film (Fisher Biotec) and were centrifuged immediately prior to analysis (1000 x rpm, 2 min) to draw down all liquid and break any bubbles. A standard melt curve analysis was conducted using a temperature ramp from 25°C to 99°C at a rate of 0.05°C/s. Fluorescence was detected using the in built ‘m4’ filter set (excitation λ 470±15 nm, emission λ 586±10 nm) and data were analyzed using Prism Software (v6.0a, GraphPad). *Tm* was determined by fitting a Boltzmann equation to the data.

### Isothermal titration calorimetry

Oxidized EcDsbA was diluted to 50 μM in 25 mM HEPES pH 7.4, 50 mM NaCl and 5% DMSO. Compounds were dissolved in 100% DMSO, and then diluted to a final concentration of 2 mM into 25 mM HEPES pH 7.4 50 mM NaCl with a final DMSO concentration of 5%. All experiments were performed using the MicroCal Auto-ITC200 instrument (GE Healthcare, USA), loading 200 μL of EcDsbA in the sample cell and 40 μL of compound in the syringe. Titrations were set up at 25°C with 19 injections of 2 μL separated by 180 seconds and a constant stirring speed of 1000 rpm. A preliminary injection of 0.5 μL was added to avoid slow leakage of titrant into the sample cell before the first 2 μL injection and the corresponding data point was excluded from analysis. Every compound was tested with three technical replicates. An additional titration of compound into buffer only (25 mM HEPES pH 7.4, 50 mM NaCl and 5% DMSO) was performed to measure the background heat of dilution. Analysis was performed with the MicroCal Origin software (version 7.0552).

### Enzyme assay

Lyophilized EcDsbA synthetic substrate (peptide CQQGFDGTQNSCK) which contains a C-terminal methylcoumarin and an N-terminal 1,4,7,10-tetraazacyclododecane-1,4,7,10-tetraacetic acid (DOTA) group (Anaspec, USA) was dissolved in 100 mM imidazole pH 6.0. 100 mM of europium trifluoromethanesulfonate (Sigma Aldrich, Australia) was added to the synthetic substrate peptide at a molar ratio of 2:1 and incubated for 5 min at room temperature to allow europium chelation. Experiments were performed in white 384-well plates (Perkin Elmer OptiPlate-384, Part #: 6007290) in 50 mM MES, 50 mM NaCl and 2 mM EDTA pH 5.5 buffer. Each well contained 80 nM EcDsbA, 1.6 μM EcDsbB, one of the tested compounds at concentrations ranging from 16 μM to 2 mM and 10 μM synthetic substrate peptide (added last to initiate the reaction) for a final volume of 50 μL per well. Positive controls were set up in which compound was replaced with buffer, and negative controls lacked EcDsbA or EcDsbB. Fluorescence emitted by substrate folding was measured with a Synergy H1 multimode plate reader (excitation λ = 340 nm and emission λ = 615 nm). Analysis was performed using the Prism software (v6.0a, GraphPad). A very low compound concentration value was used (1 nM) to allow plotting the activity of the positive control (the native EcDsbA activity without compound) on a logarithmic scale of compound concentration.

## Results

### Virtual screening and compound synthesis

**Fig 3:**
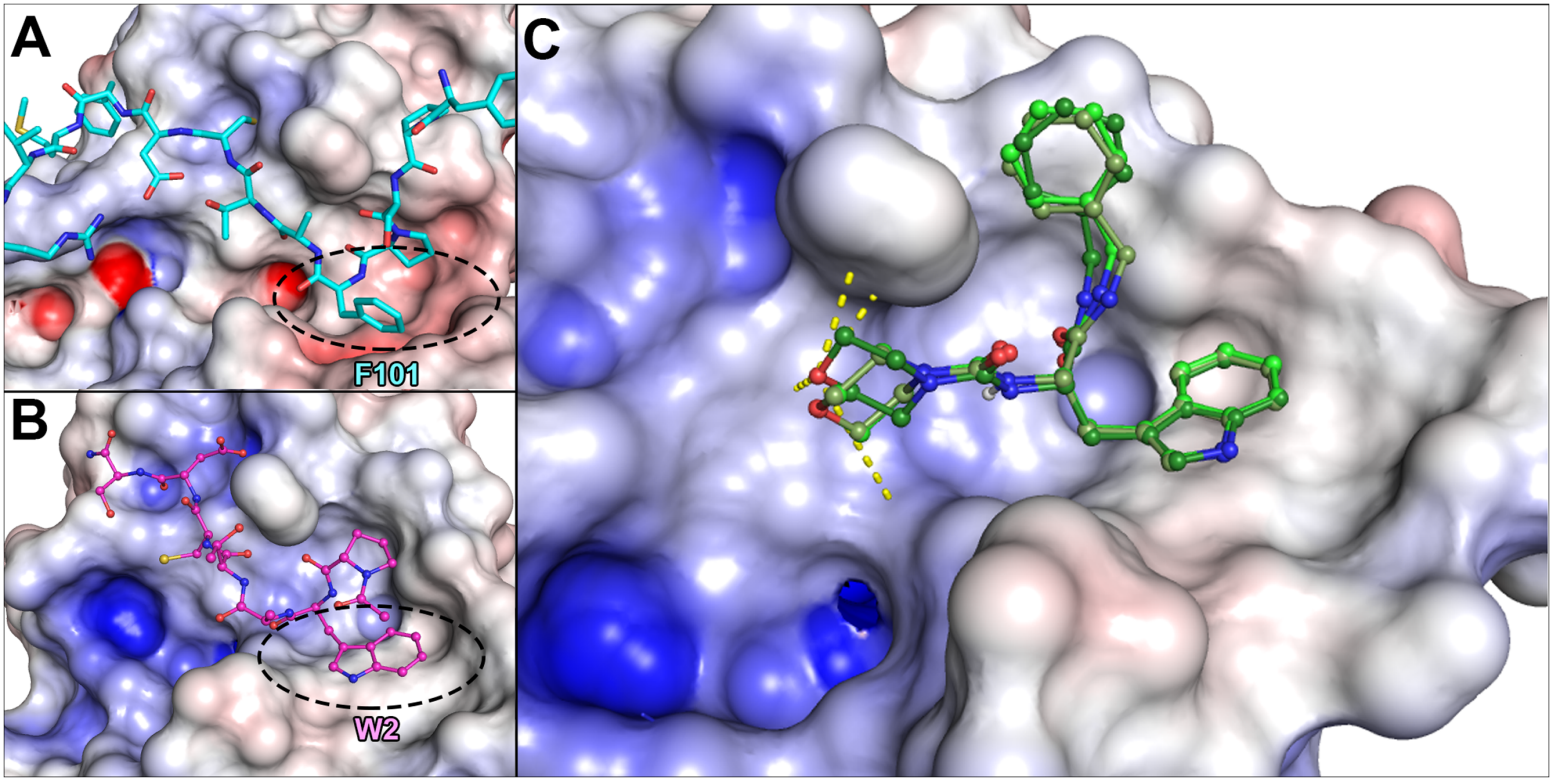
Comparison of the docked designed peptidomimetic with the EcDsbA-EcDsbB and PmDsbA-PWATCDS crystal structures. Calculated electrostatic surfaces of the enzymes are shown, with acidic regions in red, basic regions in blue and non-polar (hydrophobic) regions in white. Electrostatics cut-offs used are +/- 7.5 keV. **A.** Detail of the EcDsbA complex with EcDsbB from the crystal structure (PDB code 2ZUP [37]) centred on the ^97^YPSPFATCDFMVR^109^ sequence of EcDsbB (in light blue) showing Phe101 (F101) binding in the EcDsbA hydrophobic groove (circled). **B.** Detail of the PmDsbAC30S:PWATCDS crystal structure (PDB code 4OD7) with PWATCDS in magenta. Residue Trp2 (W2) of the peptide binds in the PmDsbA hydrophobic groove (circled). **C.** Virtual screening identified compound **1** as a potential hit. Three optimal conformations of **1** are shown (in differing shades of green), in their predicted binding mode to the PmDsbAC30S hydrophobic groove. Potential hydrogen bonds between the morpholine moiety and DsbA Pro150, His32 and Asn162 are shown as yellow dashed lines.

The 1.6 Å resolution PmDsbAC30S-PWATCDS crystal structure PDB code 4OD7 [38], rather than the 3.7 Å resolution EcDsbB-EcDsbA crystal structure (PDB 2ZUP [40]), was used to analyse the hydrophobic groove interactions (Fig 3A). Virtual screening of a peptidomimetic library was performed by focusing on the interaction of Trp2 in heptapeptide PWATCDS with Tyr173 in PmDsbAC30S within the hydrophobic groove (Fig 3B). The compound with the best fit, hereafter named compound **1**, was a tryptophan residue flanked by a C-terminal morpholine functional group and an N-terminal benzyl moiety (Fig 3C). Apart from the pi-stacking interaction between the Trp indole and Tyr173 of PmDsbAC30S, the ligand docking model showed the oxygen atom of the morpholine group at similar distances from the PmDsbAC30S Pro150 backbone amide, His32 imidazole ring amines, and the Asn162 side chain amide (≈ 4.5 Å). Compound **1** scored well in docking experiments performed using three different programs Goldscore, Chemscore and ChemPLP. Secondly, it had a good balance between aqueous solubility and "drug-like" properties (CLogP = 2.6, 6 rotatable bonds, 3 hydrogen bond acceptors, 3 hydrogen bond donors, MW = 406 Da, and is neutral at physiological pH). Compound **1** was an attractive first target for synthesis because it was predicted to extend its indole ring into the Trp-binding pocket, form hydrophobic interactions from its benzylamide group with the same region as the proline of the original peptide, and insert its morpholine ring into the groove.

**Fig 4:**
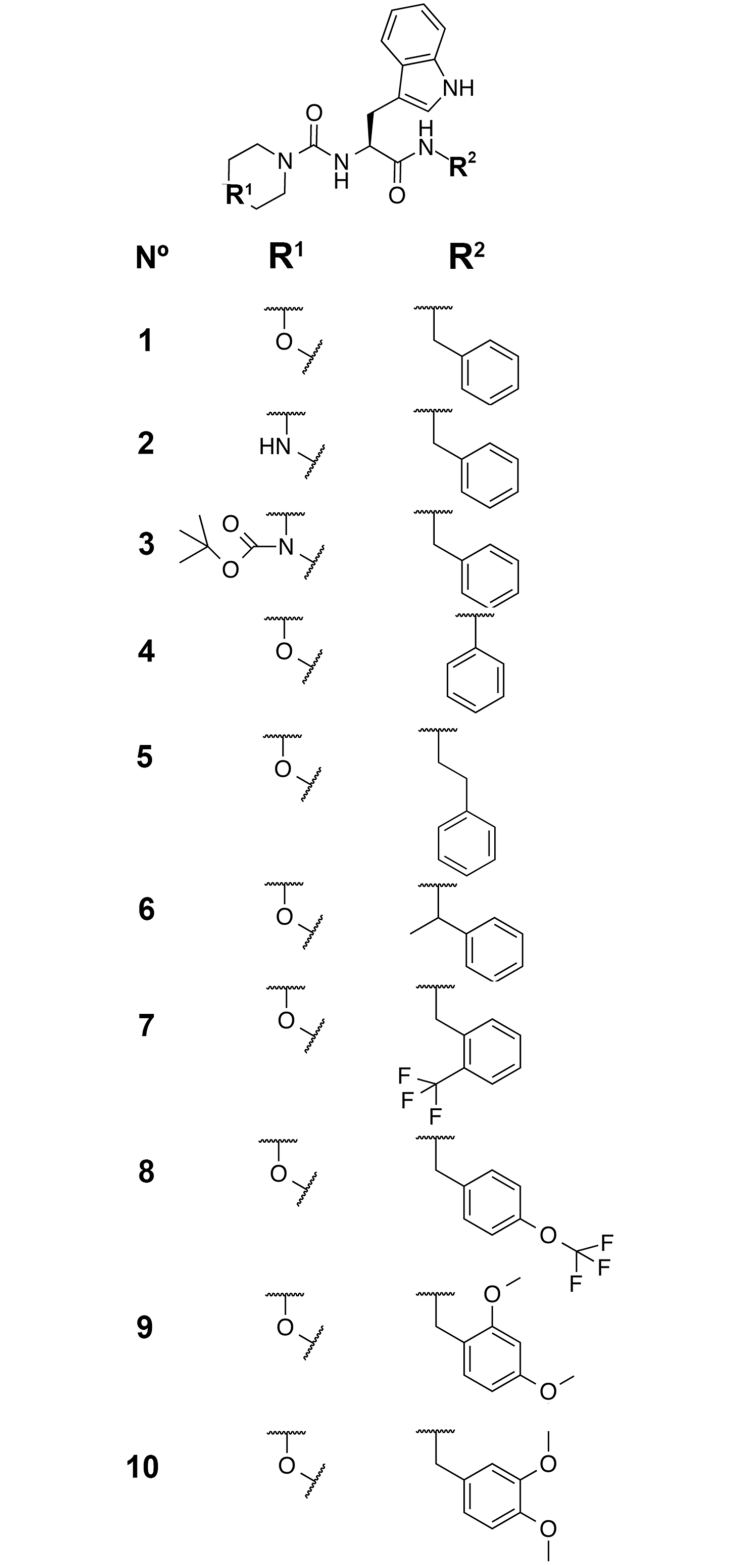
Chemical structures of the 10 peptidomimetic compounds synthesized and tested in this work. Compound **1** is the hit from the virtual screening from which derivatives **2**–**10** were designed.

Nine derivatives of **1** were also synthesized (Fig 4) to explore the binding groove and exploit potential optimization possibilities. N-Boc-tryptophan was coupled with a variety of amines using carbonyliimidazole and then deprotected using trifluoroacetic acid and anisole to give free amines at room temperature. This was subjected to treatment with carbonyldiimidazole to afford the activated carbonyl imidazole urea intermediate which reacted smoothly with morpholine or 1-Boc-piperazine at room temperature to afford compounds **1**, and **3–10**. The N-Boc of compound **3** was removed with trifluoroacetic acid to afford compound **2**.

### Differential scanning fluorimetry

The peptidomimetic compounds were first evaluated using differential scanning fluorimetry (DSF) to detect binding to EcDsbA through a concentration-dependent increase (Δ*Tm*) of EcDsbA melting temperature *Tm*. The peptidomimetics were tested over a concentration range from 64 μM to 2 mM and checked for potential compound-dye interferences that could lead to unwanted background fluorescence. When mixed with SYPRO orange on a temperature gradient from 25°C to 99°C, compounds **7** and **8** indicated strong background fluorescence at 0.5 mM, 1 mM and 2 mM concentrations. Compounds **1**, **3**, **4** and **5** showed some interaction with the dye at the highest concentration (2 mM) but the resulting fluorescence decreased to background level well before the DsbA unfolding point (<50°C), and thus would not be expected to interfere with the analysis. Finally, compounds **2**, **6**, **9**, and **10** did not demonstrate substantial fluorescence from interaction with the dye. For each compound, concentrations that demonstrated interference with the dye were excluded from the following binding experiments with EcDsbA.

**Fig 5:**
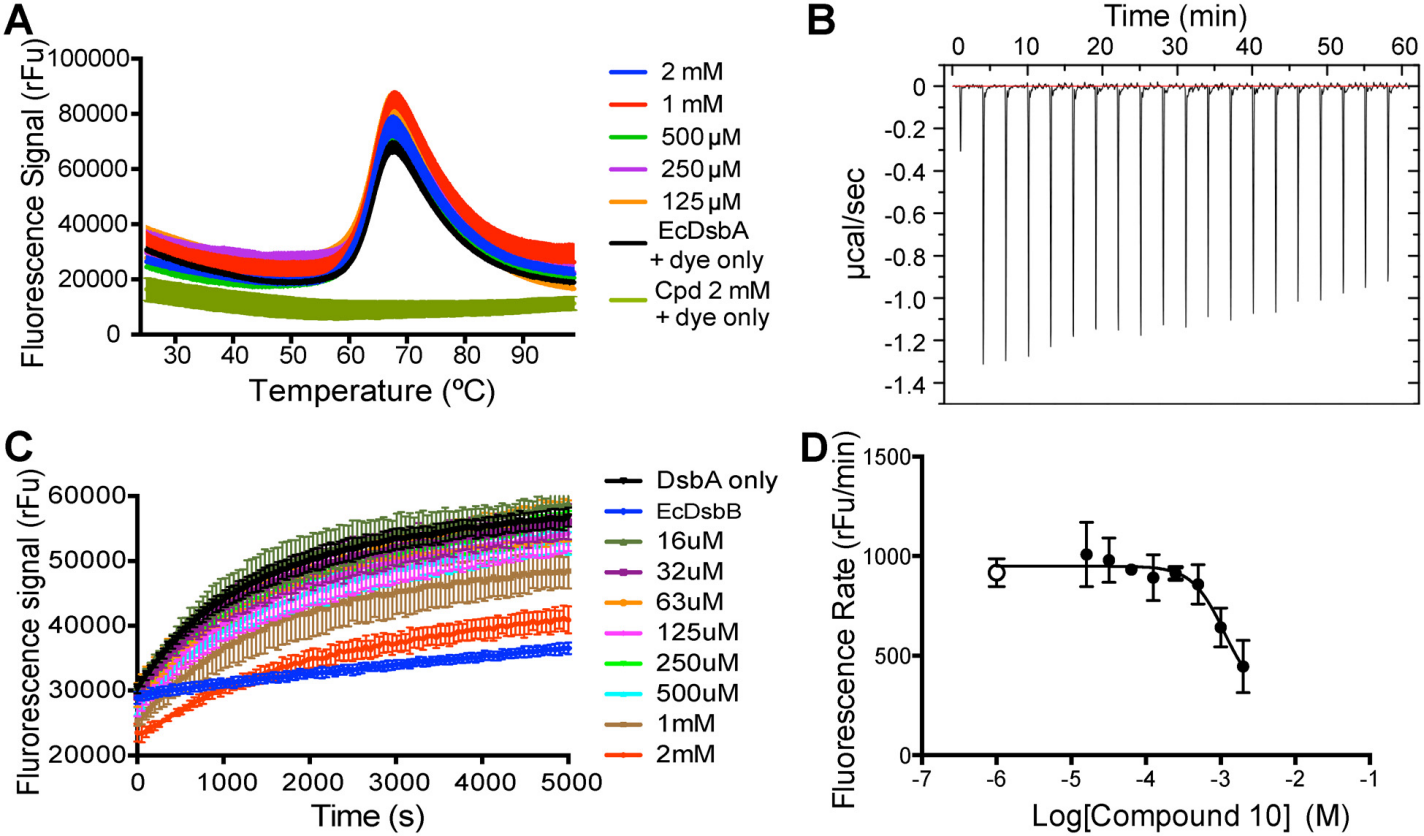
Compound 10 demonstrated weak inhibitory activity. **A.** Differential scanning fluorimetry profile with increasing concentrations of compound **10**. Similar to all other compounds tested, there was no significant shift in the unfolding temperature of EcDsbA up to 2 mM of compound **10**. **B.** ITC profile of EcDsbA titration by compound **10**, which shows no detectable binding under the conditions used (see methods for details). A similar outcome was found for the other 9 compounds. **C.** Compound **10** was the only one of the ten tested peptidomimetics that exhibited detectable activity in the DsbA assay, inducing a reduction in DsbA folding activity. **D.** Plotting the log of the peptidomimetic concentration against the rate of fluorescence increase measured in the enzyme assay allowed fitting of a sigmoidal curve and an estimated IC_50_ value of ~1 mM for compound **10**. The positive control with no compound is shown as a white circle.

To be considered significant, a binding-induced thermal shift value Δ*Tm* should be at least twice that of the estimated standard deviation of the native protein *Tm* [52], in this case 0.1°C. We found that the measured Δ*Tm* values were less than 0.2°C, and the compounds also failed to demonstrate a concentration-dependent effect on *Tm* (Fig 5A). These data suggested that the peptidomimetic compounds did not bind tightly to EcDsbA. However, DSF results can be biased by the intrinsic affinity of the compounds for the dye, by virtue of their hydrophobicity. To further investigate the potential of this series of peptidomimetic, their affinity was also evaluated using a label-free approach, namely isothermal titration calorimetry.

### Isothermal titration calorimetry

The affinity and thermodynamics of the 10 compounds were measured using ITC allowing comparison with the original peptide sequence PWATCDS (*K*d 3.0±0.1 μM, ΔH -14.3±0.5 kcal.mol^-1^, ΔH -22.6±2.8 cal.mol^-1^.deg^-1^ for EcDbsA [42] and *K*d 8.3±0.4 μM, ΔH -13.7±0.3 kcal.mol^-1^, ΔS -22.3±1.1 cal.mol^-1^.deg^-1^ for PmDsbA [38]). Titration of EcDsbA with compound **1** revealed little evidence of binding (low release of energy -0.60 μcal.sec^-1^ for the highest peak) or EcDsbA saturation. Similarly for derivatives **2**–**10** no substantial binding was detected under the conditions used (Fig 5B). The ITC profiles were similar to the negative controls, suggesting that the compounds did not bind to or had at best weak (millimolar range) affinity for EcDsbA. The low ITC signals could also result from a low enthalpic contribution to binding which might be a consequence of ligand interaction with a hydrophobic binding site [53]. We therefore assessed the ability of the compounds to inhibit the enzymatic activity of EcDsbA.

### Enzyme assay

EcDsbA activity can be assessed using a synthetic substrate and a fluorescent assay [50, 54] Inhibition of EcDsbA activity is demonstrated by a reduction in the rate of fluorescence signal emission. Inhibition by the peptidomimetics could be due to competition with the EcDsbA-substrate interaction, the EcDsbA-EcDsbB interaction or the EcDsbB-ubiquinone electron exchange. None of the compounds **1**–**9** showed any effect on EcDsbA activity at the concentrations used. However, compound **10** exhibited a reduction in fluorescence signal at high concentrations (1 mM and 2 mM, Fig 5C). Plotting the concentration versus the rate of fluorescence increase enabled an IC_50_ of 1.1 mM to be estimated for compound **10** (Fig 5D). This value is ~200-fold lower than the value for the 9-residue peptide from native EcDsbB (PSPFATCDF, 6.7 ± 1.1 μM, [42]) or the optimized 7-residue peptide PWATCDS (5.7 ± 0.4 μM [42]) for EcDsbA. Taken together, the data we present here indicate that these much smaller peptidomimetic compounds were poor inhibitors of EcDsbA activity with IC_50_ values estimated in the millimolar range.

## Discussion

The antibiotic pipeline is in urgent need of new antibacterials especially for Gram-negative pathogens. EcDsbA is a potential antibacterial target because it catalyzes the assembly of virulence factors. The present work is part of an effort towards the development of EcDsbA inhibitors. It follows up on the observation that a peptide PWATCDS binds non-covalently to a cysteine mutant of *P*. *mirabilis* DsbA (PmDsbAC30S), making interactions with the hydrophobic groove of the enzyme [38]. The series of peptidomimetic fragments described here was designed to target the hydrophobic peptide- and DsbB-binding groove of the EcDsbA protein. Peptidomimetics have been highlighted as an important source of novel antimicrobial scaffolds [55–62]. They offer the advantage of being able to mimic protein-protein interactions, but having better properties as drug candidates than peptides (improved stability and pharmacokinetics, lower immunogenicity, lower molecular mass [63–67]).

To start this line of fragment-focused research, we employed virtual screening of the PmDsbAC30S-PWATCDS crystal structure, focusing on the hydrophobic groove fitted by the 7-residue peptide, and identified a virtual hit fragment from a peptidomimetic library (compound **1**), around which nine derivatives were also synthesized (compounds **2**–**10**). No evidence of binding was detected by differential scanning fluorimetry or by ITC, but a functional enzyme assay indicated weak inhibition of EcDsbA by compound **10** (IC_50_ 1.1 mM). Although these small fragment compounds had > 200-fold reduction in affinity and inhibition of EcDsbA compared with the much larger 7-residue *covalent* peptide PWATCDS, they might represent useful templates for rationally deriving more drug-like small organic and peptidomimetic compounds to bind *non-covalently* to the enzyme.

Three possibilities could account for these findings. First, these small peptidomimetics built around tryptophan may simply not bind well to the DsbA hydrophobic groove, perhaps due to being so small or conformationally restricted. Second, the small peptidomimetics bind weakly and reversibly to the hydrophobic groove, and are easily displaced by substrate or DsbB. This possibility would imply that the hydrophobic contacts formed between the peptidomimetic and the EcDsbA groove are insufficient in number or too weak to compete with the longer peptide substrate or the protein substrate EcDsbB, both of which also make an additional covalent interaction with the EcDsbA active site cysteine. Third, the peptidomimetic binds tightly to the groove (but is not detected by DSF because of interference, or by ITC because of a major entropic component to binding), and disulfide bond transfer occurs between EcDsbA/substrate and EcDsbA/EcDsbB despite the hydrophobic groove interaction. The role of the hydrophobic groove as the sole binding interaction for peptidomimetic drugs may therefore be limited in this system. However, inhibitors of DsbA-mediated cellular motility in *E*. *coli* have recently been identified using a fragment-screening approach that bind to the hydrophobic groove with a Kd of 200 μM [68]. We therefore consider that the second of the three explanations is probably true, and that additional interactions are needed for inhibitor potency. In the future, it may be possible to combine such inhibitors with other peptidomimetic fragments that interact with enzyme active site regions, the C*XX*C motif or the cisPro loop to realize the necessary drug-like potencies being sought.

## Supporting Information

S1 Fig NMR spectral data of compound 1 (*S*)-2-[*N*-(Morpholine-1-carbonyl)amino]-3-(1*H*-indol-3-yl)-*N*-(benzyl)propanamide. ^1^H NMR (400 MHz, CDCl_3_): δ 7.96 (br, 1H), 7.72 (d, *J* = 7.8 Hz, 1H), 7.36 (d, *J* = 7.8 Hz, 1H), 7.23–7.19 (m, 5H), 7.13(t, *J* = 6.6 Hz, 1H), 6.99 (d, *J* = 7.8 Hz, 1H), 6.93 (br, 1H), 5.99 (s, 1H), 5.28 (d, *J* = 7.8 Hz, 1H), 4.68–4.64 (m, 1H), 4.32 (dd, *J* = 13.2, 4.8 Hz, 1H), 4.24 (dd, *J* = 13.2, 4.8 Hz, 1H), 3.64–3.59 (m, 4H), 3.37 (dd, *J* = 13.2, 6.6 Hz, 1H), 3.33–3.30 (m, 2H), 3.26–3.22 (m, 2H), 3.13(d, *J* = 12.6 Hz, 1H). ^13^C NMR (101 MHz, CDCl_3_): δ 172.03, 157.09, 137.71, 136.16, 128.55, 127.63, 127.41, 123.12, 122.49, 119.88, 118.82, 111.28, 110.93, 66.46, 55.31, 43.95, 43.58, 28.89. HRMS (ESI^+^): C_23_H_27_N_4_O_3_^+^ [MH]^+^ calcd: 407.2078, found: 407.2079.
(PNG)

S2 Fig NMR spectral data of compound 2 (*S*)-2-[*N*-(Piperazine-1-carbonyl)amino]-3-(1*H*-indol-3-yl)-*N*-(benzyl)propanamide. ^1^H NMR (600 MHz, DMSO-*d*_*6*_): δ 10.82 (br, 1H), 8.79 (br, 1H), 8.47 (t, *J* = 6.6 Hz, 1H), 7.64 (d, *J* = 7.8 Hz, 1H), 7.34 (d, *J* = 7.8 Hz, 1H), 7.3 (t, *J* = 7.8 Hz, 2H), 7.21 (t, *J* = 7.8 Hz, 1H), 7.16 (t, *J* = 7.2 Hz, 3H), 7.06 (t, *J* = 7.2 Hz, 1H), 6.97 (t, *J* = 7.8 Hz, 1H), 6.81 (d, *J* = 7.8 Hz, 1H), 4.44–4.40 (m, 1H), 4.32–4.25 (m, 2H), 3.51–3.42 (m, 4H), 3.13 (dd, *J* = 14.4, 4.8 Hz, 1H), 3.02–2.98 (m, 5H). ^13^C NMR (151 MHz, DMSO-*d*_*6*_): δ 172.57, 156.70, 139.39, 136.06, 128.16, 127.37, 126.95, 126.58, 123.75, 120.79, 118.58, 118.12, 111.27, 110.59, 55.56, 42.57, 41.98, 40.66, 27.91. HRMS (ESI^+^): C_23_H_28_N_5_O_2_^+^ [MH]^+^ calcd: 406.2238, found: 406.2233.
(PNG)

S3 Fig NMR spectral data of compound 3 (*S*)-2-[*N*-(*tert*-Butyl-4-piperazine-1-carbonyl)amino]-3-(1*H*-indol-3-yl)-*N*-(benzyl)propanamide. ^1^H NMR (600 MHz, CDCl_3_): δ 8.07 (br, 1H), 7.65 (d, *J* = 8.4 Hz, 1H), 7.36 (d, *J* = 7.8 Hz, 1H), 7.24–7.21(m, 3H), 7.20 (t, *J* = 7.8 Hz, 1H), 7.10 (t, *J* = 7.8 Hz, 1H), 6.97 (dd, *J* = 7.0, 3.0 Hz, 2H), 6.93 (d, *J* = 1.8 Hz, 1H), 6.42 (br, 1H), 5.45 (d, *J* = 6.6 Hz, 1H), 4.69 (q, *J* = 7.8 Hz, 1H), 4.34 (dd, *J* = 15.6, 6.6 Hz, 1H), 4.23 (dd, *J* = 15.6, 6.6 Hz, 1H), 3.31–3.14 (m, 10H), 1.47 (m, 9H). ^13^C NMR (151 MHz, CDCl_3_): δ 172.48, 156.95, 154.59, 137.45, 136.15, 128.55, 127.62, 127.44, 127.35, 123.22, 122.43, 119.90, 118.70, 111.31, 110.68, 80.36, 55.46, 43.61, 43.40, 28.70, 28.35. HRMS (ESI^+^): C_28_H_36_N_5_O_4_^+^ [MH]^+^ calcd: 506.2762, found: 506.2761.
(PNG)

S4 Fig NMR spectral data of compound 4 (*S*)-2-[*N*-(Morpholine-1-carbonyl)amino]-3-(1*H*-indol-3-yl)-*N*-(phenyl)propanamide. ^1^H NMR (400 MHz, CDCl_3_): δ 8.09 (br, 1H), 7.88 (br, 1H), 7.75 (d, *J* = 7.2 Hz, 1H), 7.39 (d, *J* = 7.8 Hz, 1H), 7.30–7.21 (m, 3H), 7.16–7.12 (m, 2H), 7.06 (t, *J* = 7.2 Hz, 1H), 5.27 (d, *J* = 7.8 Hz, 1H), 4.78 (q, *J* = 7.8 Hz, 1H), 3.64–3.57 (m, 4H), 3.47 (dd, *J* = 14.4, 5.4 Hz, 1H), 3.33–3.20 (m, 5H). ^13^C NMR (151 MHz, CDCl_3_): δ 170.34, 157.36, 137.40, 136.22, 128.93, 127.42, 124.39, 123.40, 122.62, 120.10, 119.94, 118.82, 111.42, 110.93, 66.40, 55.83, 44.02, 28.04. HRMS (ESI^+^): C_22_H_25_N_4_O_3_^+^ [MH]^+^ calcd: 393.1921, found: 393.1921.
(PNG)

S5 Fig NMR spectral data of compound 5 (*S*)-2-[*N*-(Morpholine-1-carbonyl)amino]-3-(1*H*-indol-3-yl)-*N*-(phenethyl)propanamide. ^1^H NMR (600 MHz, CDCl_3_): δ 8.07 (br, 1H), 7.67 (d, *J* = 7.8 Hz, 1H), 7.37 (d, *J* = 7.8 Hz, 1H), 7.21(t, *J* = 7.8 Hz, 1H), 7.16–7.12 (m, 4H), 6.96 (d, *J* = 2.4 Hz, 1H), 6.89–6.87 (m, 2H), 5.79 (br, 1H), 5.29 (d, *J* = 7.8 Hz, 1H), 4.60–4.56 (m, 1H), 3.59 (t, *J* = 5.4 Hz, 4H), 3.42–3.20 (m, 7H), 3.09 (dd, *J* = 15.6, 9.0 Hz, 1H), 2.60–2.55 (m, 1H), 2.51–2.46 (m, 1H). ^13^C NMR (151 MHz, CDCl_3_): δ 172.18, 157.01, 138.49, 136.18, 128.60, 128.54, 127.46, 126.47, 123.18, 122.49, 119.92, 118.84, 111.38, 110.97, 66.38, 55.33, 43.87, 40.53, 35.28, 28.68. HRMS (ESI^+^): C_24_H_29_N_4_O_3_^+^ [MH]^+^ calcd: 421.2234, found: 421.2232.
(PNG)

S6 Fig NMR spectral data of compound 6 (*S*)-2-[*N*-(Morpholine-1-carbonyl)amino]-3-(1*H*-indol-3-yl)-*N*-(1-phenylethyl)propanamide. ^1^H NMR (600 MHz, CDCl_3_): δ 7.77 (br, 1H), 7.71 (d, *J* = 8.4 Hz, 1H), 7.31 (d, *J* = 8.4 Hz, 1H), 7.24–7.23 (m, 2H), 7.18 (t, *J* = 7.8 Hz, 1H), 7.12 (t, *J* = 7.8 Hz, 1H), 7.24–7.23 (m, 2H), 6.67 (d, *J* = 2.4 Hz, 1H), 5.92 (d, *J* = 7.8 Hz, 1H), 5.40 (d, *J* = 7.8 Hz, 1H), 4.97 (q, *J* = 6.0 Hz, 1H), 4.66–4.62 (m, 1H), 3.63–3.59 (m, 4H), 3.34–3.25 (m, 5H), 3.04 (dd, *J* = 15.0, 9.0 Hz, 1H), 1.33 (d, *J* = 7.2 Hz, 3H). ^13^C NMR (151 MHz, CDCl_3_): δ 171.23, 157.10, 142.72, 136.12, 128.54, 127.32, 127.20, 126.11, 123.27, 122.38, 119.90, 118.99, 111.24, 110.76, 66.37, 54.94, 49.02, 43.94, 29.13, 21.58. HRMS (ESI^+^): C_24_H_29_N_4_O_3_^+^ [MH]^+^ calcd: 421.2234, found: 421.2231.
(PNG)

S7 Fig NMR spectral data of compound 7 (*S*)-2-[*N*-(Morpholine-1-carbonyl)amino]-3-(1*H*-indol-3-yl)-*N*-(2-(trifluoromethyl)benzyl)propanamide. ^1^H NMR (600 MHz, CDCl_3_): δ 8.01 (br, 1H), 7.62 (d, *J* = 7.8 Hz, 1H), 7.58 (d, *J* = 7.2 Hz, 1H), 7.38 (t, *J* = 7.8 Hz, 1H), 7.35–7.31 (m, 2H), 7.19 (t, *J* = 7.2 Hz, 1H), 7.14 (d, *J* = 8.4 Hz, 1H), 7.09 (t, *J* = 7.2 Hz, 1H), 6.90 (d, *J* = 1.8 Hz, 1H), 6.45 (br, 1H), 5.44 (br, 1H), 4.73 (q, *J* = 6.6 Hz, 1H), 4.51 (dd, *J* = 15.6, 6.0 Hz, 1H), 4.44 (dd, *J* = 15.6, 6.0 Hz, 1H), 3.60–3.56 (m, 4H), 3.32–3.13 (m, 6H). ^13^C NMR (151 MHz, CDCl_3_): δ 172.70, 157.22, 136.20, 135.88, 132.15, 129.96, 127.98 (q, *J* = 30.2 Hz), 127.51, 127.26, 125.89 (q, *J* = 5.9 Hz), 124.29 (q, *J* = 272 Hz), 123.11, 122.48, 119.94, 118.61, 111.30, 110.50, 66.28, 55.30, 43.89, 40.10, 28.53. HRMS (ESI^+^): C_24_H_26_F_3_N_4_O_3_^+^ [MH]^+^ calcd: 475.1952, found: 475.1951.
(PNG)

S8 Fig NMR spectral data of compound 8 (*S*)-2-[*N*-(Morpholine-1-carbonyl)amino]-3-(1*H*-indol-3-yl)-*N*-(3-(trifluoromethoxy)benzyl)propanamide. ^1^H NMR (600 MHz, CDCl_3_): δ 8.02 (br, 1H), 7.65 (d, *J* = 8.4 Hz, 1H), 7.36 (d, *J* = 8.4 Hz, 1H), 7.24 (t, *J* = 6.6 Hz, 1H), 7.20 (t, *J* = 6.6 Hz, 1H), 7.12 (t, *J* = 7.8 Hz, 1H), 7.07 (d, *J* = 7.8 Hz, 1H), 6.97 (s, 1H), 6.91 (d, *J* = 7.8 Hz, 1H), 6.84 (s, 1H), 6.45 (br, 1H), 5.44 (br, 1H), 4.71–4.68 (m, 1H), 4.32 (dd, *J* = 15.6, 6.6 Hz, 1H), 4.25 (dd, *J* = 15.6, 6.6 Hz, 1H), 3.63–3.57 (m, 4H), 3.34–3.14 (m, 6H). ^13^C NMR (151 MHz, CDCl_3_): δ 172.79, 157.27, 149.38, 139.97, 136.21, 129.98, 127.27, 125.92, 123.15 (q, 270.1 Hz), 122.57, 121.26, 120.01, 119.96, 119.76, 118.62, 111.40, 110.58, 66.31, 55.44, 43.90, 42.99, 28.56. HRMS (ESI^+^): C_24_H_26_F_3_N_4_O_4_^+^ [MH]^+^ calcd: 491.1901, found: 491.1902.
(PNG)

S9 Fig NMR spectral data of compound 9 (*S*)-2-[*N*-(Morpholine-1-carbonyl)amino]-3-(1*H*-indol-3-yl)-*N*-(2,4-(dimethoxybenzyl)propanamide. ^1^H NMR (400 MHz, CDCl_3_): δ 7.91 (br, 1H), 7.59 (d, *J* = 8.6 Hz, 1H), 7.28 (d, *J* = 10.4 Hz, 1H), 7.14(t, *J* = 10.4 Hz, 1H), 7.08(t, *J* = 10.4 Hz, 1H), 6.92 (d, *J* = 10.4 Hz, 1H), 6.77 (s, 1H), 6.53 (s, 1H), 6.35 (d, *J* = 10.4 Hz, 1H), 6.32 (s, 1H), 5.75 (br, 1H), 4.67 (br, 1H), 4.27 (dd, *J* = 13.8, 6.8 Hz, 1H), 4.14 (dd, *J* = 13.8, 6.8 Hz, 1H), 3.79 (s, 3H), 3.59 (t, *J* = 4.7 Hz, 4H), 3.55 (s, 3H), 3.29–3.19 (m, 5H), 3.11–3.04 (m, 1H). ^13^C NMR (101 MHz, CDCl_3_): δ 172.49, 160.56, 158.39, 157.21, 136.11, 130.22, 127.12, 127.11, 123.30, 122.21, 119.75, 118.62, 117.82, 111.16, 110.13, 103.73, 98.39, 98.35, 66.29, 55.44, 54.97, 44.90, 39.31, 28.90. HRMS (ESI^+^): C_25_H_31_N_4_O_5_^+^ [MH]^+^ calcd: 467.2289, found: 467.2287.
(PNG)

S10 Fig NMR spectral data of compound 10 *S*)-2-[*N*-(Morpholine-1-carbonyl)amino]-3-(1*H*-indol-3-yl)-*N*-(2,4-(dimethoxybenzyl)propanamide. ^1^H NMR (400 MHz, CDCl_3_): δ 8.04 (br, 1H), 7.62 (d, *J* = 6.4 Hz, 1H), 7.34 (d, *J* = 8.6 Hz, 1H), 7.20 (t, *J* = 6.6 Hz, 1H), 7.12 (d, *J* = 7.6 Hz, 1H), 6.92 (s, 1H), 6.72 (d, *J* = 8.5 Hz, 1H), 6.59 (s, 1H), 6.55 (d, *J* = 9.48 Hz, 1H), 6.40 (br, 1H), 5.60 (br, 1H), 4.67 (s, 1H), 4.29–4.16 (m, 3H), 3.86 (s, 3H), 3.79 (s, 1H), 3.61 (t, *J* = 4.2 Hz, 4H), 3.32–3.13 (m, 6H). ^13^C NMR (101 MHz, CDCl_3_): δ 172.72, 157.32, 149.06, 148.51, 136.24, 132.67, 129.80, 127.33, 123.26, 122.54, 120.06, 119.90, 118.63, 111.29, 111.22, 111.18, 111.10, 110.49, 66.25, 55.98, 55.57, 43.91, 43.60, 28.64. HRMS (ESI^+^): C_25_H_31_N_4_O_5_^+^ [MH]^+^ calcd: 467.2289, found: 467.2287.
(PNG)
