# Supplementary figures and images for: Virtual Screening of Peptide and Peptidomimetic Fragments Targeted to Inhibit Bacterial Dithiol Oxidase DsbA
Source: PLoS One. 2015 Jul 30;10(7):e0133805. doi: 10.1371/journal.pone.0133805 (PMC4520593; doi:10.1371/journal.pone.0133805)

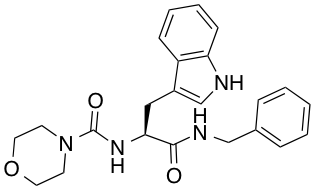

Supplement: S1 Fig — 1H NMR (400 MHz, CDCl3): δ 7.96 (br, 1H), 7.72 (d, J = 7.8 Hz, 1H), 7.36 (d, J = 7.8 Hz, 1H), 7.23–7.19 (m, 5H), 7.13(t, J = 6.6 Hz, 1H), 6.99 (d, J = 7.8 Hz, 1H), 6.93 (br, 1H), 5.99 (s, 1H), 5.28 (d, J = 7.8 Hz, 1H), 4.68–4.64 (m, 1H), 4.32 (dd, J = 13.2, 4.8 Hz, 1H), 4.24 (dd, J = 13.2, 4.8 Hz, 1H), 3.64–3.59 (m, 4H), 3.37 (dd, J = 13.2, 6.6 Hz, 1H), 3.33–3.30 (m, 2H), 3.26–3.22 (m, 2H), 3.13(d, J = 12.6 Hz, 1H). 13C NMR (101 MHz, CDCl3): δ 172.03, 157.09, 137.71, 136.16, 128.55, 127.63, 127.41, 123.12, 122.49, 119.88, 118.82, 111.28, 110.93, 66.46, 55.31, 43.95, 43.58, 28.89. HRMS (ESI+): C23H27N4O3 + [MH]+ calcd: 407.2078, found: 407.2079. (PNG) [file pone.0133805.s001.png]

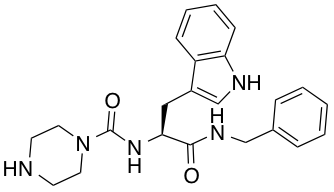

Supplement: S2 Fig — 1H NMR (600 MHz, DMSO-d 6): δ 10.82 (br, 1H), 8.79 (br, 1H), 8.47 (t, J = 6.6 Hz, 1H), 7.64 (d, J = 7.8 Hz, 1H), 7.34 (d, J = 7.8 Hz, 1H), 7.3 (t, J = 7.8 Hz, 2H), 7.21 (t, J = 7.8 Hz, 1H), 7.16 (t, J = 7.2 Hz, 3H), 7.06 (t, J = 7.2 Hz, 1H), 6.97 (t, J = 7.8 Hz, 1H), 6.81 (d, J = 7.8 Hz, 1H), 4.44–4.40 (m, 1H), 4.32–4.25 (m, 2H), 3.51–3.42 (m, 4H), 3.13 (dd, J = 14.4, 4.8 Hz, 1H), 3.02–2.98 (m, 5H). 13C NMR (151 MHz, DMSO-d 6): δ 172.57, 156.70, 139.39, 136.06, 128.16, 127.37, 126.95, 126.58, 123.75, 120.79, 118.58, 118.12, 111.27, 110.59, 55.56, 42.57, 41.98, 40.66, 27.91. HRMS (ESI+): C23H28N5O2 + [MH]+ calcd: 406.2238, found: 406.2233. (PNG) [file pone.0133805.s002.png]

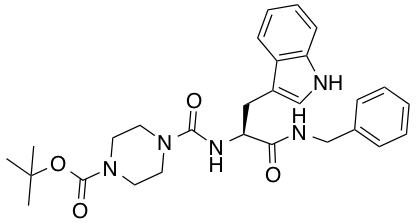

Supplement: S3 Fig — 1H NMR (600 MHz, CDCl3): δ 8.07 (br, 1H), 7.65 (d, J = 8.4 Hz, 1H), 7.36 (d, J = 7.8 Hz, 1H), 7.24–7.21(m, 3H), 7.20 (t, J = 7.8 Hz, 1H), 7.10 (t, J = 7.8 Hz, 1H), 6.97 (dd, J = 7.0, 3.0 Hz, 2H), 6.93 (d, J = 1.8 Hz, 1H), 6.42 (br, 1H), 5.45 (d, J = 6.6 Hz, 1H), 4.69 (q, J = 7.8 Hz, 1H), 4.34 (dd, J = 15.6, 6.6 Hz, 1H), 4.23 (dd, J = 15.6, 6.6 Hz, 1H), 3.31–3.14 (m, 10H), 1.47 (m, 9H). 13C NMR (151 MHz, CDCl3): δ 172.48, 156.95, 154.59, 137.45, 136.15, 128.55, 127.62, 127.44, 127.35, 123.22, 122.43, 119.90, 118.70, 111.31, 110.68, 80.36, 55.46, 43.61, 43.40, 28.70, 28.35. HRMS (ESI+): C28H36N5O4 + [MH]+ calcd: 506.2762, found: 506.2761. (PNG) [file pone.0133805.s003.png]

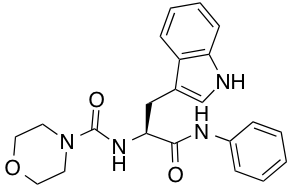

Supplement: S4 Fig — 1H NMR (400 MHz, CDCl3): δ 8.09 (br, 1H), 7.88 (br, 1H), 7.75 (d, J = 7.2 Hz, 1H), 7.39 (d, J = 7.8 Hz, 1H), 7.30–7.21 (m, 3H), 7.16–7.12 (m, 2H), 7.06 (t, J = 7.2 Hz, 1H), 5.27 (d, J = 7.8 Hz, 1H), 4.78 (q, J = 7.8 Hz, 1H), 3.64–3.57 (m, 4H), 3.47 (dd, J = 14.4, 5.4 Hz, 1H), 3.33–3.20 (m, 5H). 13C NMR (151 MHz, CDCl3): δ 170.34, 157.36, 137.40, 136.22, 128.93, 127.42, 124.39, 123.40, 122.62, 120.10, 119.94, 118.82, 111.42, 110.93, 66.40, 55.83, 44.02, 28.04. HRMS (ESI+): C22H25N4O3 + [MH]+ calcd: 393.1921, found: 393.1921. (PNG) [file pone.0133805.s004.png]

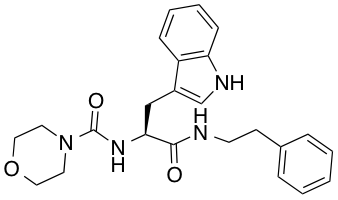

Supplement: S5 Fig — 1H NMR (600 MHz, CDCl3): δ 8.07 (br, 1H), 7.67 (d, J = 7.8 Hz, 1H), 7.37 (d, J = 7.8 Hz, 1H), 7.21(t, J = 7.8 Hz, 1H), 7.16–7.12 (m, 4H), 6.96 (d, J = 2.4 Hz, 1H), 6.89–6.87 (m, 2H), 5.79 (br, 1H), 5.29 (d, J = 7.8 Hz, 1H), 4.60–4.56 (m, 1H), 3.59 (t, J = 5.4 Hz, 4H), 3.42–3.20 (m, 7H), 3.09 (dd, J = 15.6, 9.0 Hz, 1H), 2.60–2.55 (m, 1H), 2.51–2.46 (m, 1H). 13C NMR (151 MHz, CDCl3): δ 172.18, 157.01, 138.49, 136.18, 128.60, 128.54, 127.46, 126.47, 123.18, 122.49, 119.92, 118.84, 111.38, 110.97, 66.38, 55.33, 43.87, 40.53, 35.28, 28.68. HRMS (ESI+): C24H29N4O3 + [MH]+ calcd: 421.2234, found: 421.2232. (PNG) [file pone.0133805.s005.png]

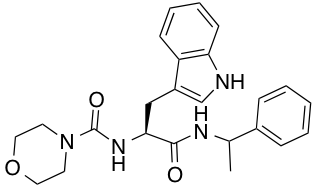

Supplement: S6 Fig — 1H NMR (600 MHz, CDCl3): δ 7.77 (br, 1H), 7.71 (d, J = 8.4 Hz, 1H), 7.31 (d, J = 8.4 Hz, 1H), 7.24–7.23 (m, 2H), 7.18 (t, J = 7.8 Hz, 1H), 7.12 (t, J = 7.8 Hz, 1H), 7.24–7.23 (m, 2H), 6.67 (d, J = 2.4 Hz, 1H), 5.92 (d, J = 7.8 Hz, 1H), 5.40 (d, J = 7.8 Hz, 1H), 4.97 (q, J = 6.0 Hz, 1H), 4.66–4.62 (m, 1H), 3.63–3.59 (m, 4H), 3.34–3.25 (m, 5H), 3.04 (dd, J = 15.0, 9.0 Hz, 1H), 1.33 (d, J = 7.2 Hz, 3H). 13C NMR (151 MHz, CDCl3): δ 171.23, 157.10, 142.72, 136.12, 128.54, 127.32, 127.20, 126.11, 123.27, 122.38, 119.90, 118.99, 111.24, 110.76, 66.37, 54.94, 49.02, 43.94, 29.13, 21.58. HRMS (ESI+): C24H29N4O3 + [MH]+ calcd: 421.2234, found: 421.2231. (PNG) [file pone.0133805.s006.png]

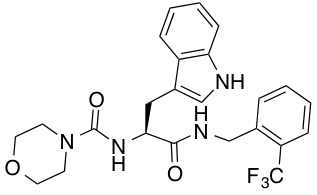

Supplement: S7 Fig — 1H NMR (600 MHz, CDCl3): δ 8.01 (br, 1H), 7.62 (d, J = 7.8 Hz, 1H), 7.58 (d, J = 7.2 Hz, 1H), 7.38 (t, J = 7.8 Hz, 1H), 7.35–7.31 (m, 2H), 7.19 (t, J = 7.2 Hz, 1H), 7.14 (d, J = 8.4 Hz, 1H), 7.09 (t, J = 7.2 Hz, 1H), 6.90 (d, J = 1.8 Hz, 1H), 6.45 (br, 1H), 5.44 (br, 1H), 4.73 (q, J = 6.6 Hz, 1H), 4.51 (dd, J = 15.6, 6.0 Hz, 1H), 4.44 (dd, J = 15.6, 6.0 Hz, 1H), 3.60–3.56 (m, 4H), 3.32–3.13 (m, 6H). 13C NMR (151 MHz, CDCl3): δ 172.70, 157.22, 136.20, 135.88, 132.15, 129.96, 127.98 (q, J = 30.2 Hz), 127.51, 127.26, 125.89 (q, J = 5.9 Hz), 124.29 (q, J = 272 Hz), 123.11, 122.48, 119.94, 118.61, 111.30, 110.50, 66.28, 55.30, 43.89, 40.10, 28.53. HRMS (ESI+): C24H26F3N4O3 + [MH]+ calcd: 475.1952, found: 475.1951. (PNG) [file pone.0133805.s007.png]

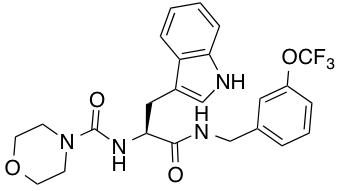

Supplement: S8 Fig — 1H NMR (600 MHz, CDCl3): δ 8.02 (br, 1H), 7.65 (d, J = 8.4 Hz, 1H), 7.36 (d, J = 8.4 Hz, 1H), 7.24 (t, J = 6.6 Hz, 1H), 7.20 (t, J = 6.6 Hz, 1H), 7.12 (t, J = 7.8 Hz, 1H), 7.07 (d, J = 7.8 Hz, 1H), 6.97 (s, 1H), 6.91 (d, J = 7.8 Hz, 1H), 6.84 (s, 1H), 6.45 (br, 1H), 5.44 (br, 1H), 4.71–4.68 (m, 1H), 4.32 (dd, J = 15.6, 6.6 Hz, 1H), 4.25 (dd, J = 15.6, 6.6 Hz, 1H), 3.63–3.57 (m, 4H), 3.34–3.14 (m, 6H). 13C NMR (151 MHz, CDCl3): δ 172.79, 157.27, 149.38, 139.97, 136.21, 129.98, 127.27, 125.92, 123.15 (q, 270.1 Hz), 122.57, 121.26, 120.01, 119.96, 119.76, 118.62, 111.40, 110.58, 66.31, 55.44, 43.90, 42.99, 28.56. HRMS (ESI+): C24H26F3N4O4 + [MH]+ calcd: 491.1901, found: 491.1902. (PNG) [file pone.0133805.s008.png]

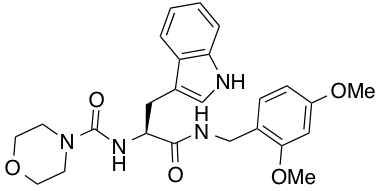

Supplement: S9 Fig — 1H NMR (400 MHz, CDCl3): δ 7.91 (br, 1H), 7.59 (d, J = 8.6 Hz, 1H), 7.28 (d, J = 10.4 Hz, 1H), 7.14(t, J = 10.4 Hz, 1H), 7.08(t, J = 10.4 Hz, 1H), 6.92 (d, J = 10.4 Hz, 1H), 6.77 (s, 1H), 6.53 (s, 1H), 6.35 (d, J = 10.4 Hz, 1H), 6.32 (s, 1H), 5.75 (br, 1H), 4.67 (br, 1H), 4.27 (dd, J = 13.8, 6.8 Hz, 1H), 4.14 (dd, J = 13.8, 6.8 Hz, 1H), 3.79 (s, 3H), 3.59 (t, J = 4.7 Hz, 4H), 3.55 (s, 3H), 3.29–3.19 (m, 5H), 3.11–3.04 (m, 1H). 13C NMR (101 MHz, CDCl3): δ 172.49, 160.56, 158.39, 157.21, 136.11, 130.22, 127.12, 127.11, 123.30, 122.21, 119.75, 118.62, 117.82, 111.16, 110.13, 103.73, 98.39, 98.35, 66.29, 55.44, 54.97, 44.90, 39.31, 28.90. HRMS (ESI+): C25H31N4O5 + [MH]+ calcd: 467.2289, found: 467.2287. (PNG) [file pone.0133805.s009.png]

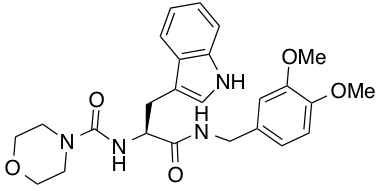

Supplement: S10 Fig — 1H NMR (400 MHz, CDCl3): δ 8.04 (br, 1H), 7.62 (d, J = 6.4 Hz, 1H), 7.34 (d, J = 8.6 Hz, 1H), 7.20 (t, J = 6.6 Hz, 1H), 7.12 (d, J = 7.6 Hz, 1H), 6.92 (s, 1H), 6.72 (d, J = 8.5 Hz, 1H), 6.59 (s, 1H), 6.55 (d, J = 9.48 Hz, 1H), 6.40 (br, 1H), 5.60 (br, 1H), 4.67 (s, 1H), 4.29–4.16 (m, 3H), 3.86 (s, 3H), 3.79 (s, 1H), 3.61 (t, J = 4.2 Hz, 4H), 3.32–3.13 (m, 6H). 13C NMR (101 MHz, CDCl3): δ 172.72, 157.32, 149.06, 148.51, 136.24, 132.67, 129.80, 127.33, 123.26, 122.54, 120.06, 119.90, 118.63, 111.29, 111.22, 111.18, 111.10, 110.49, 66.25, 55.98, 55.57, 43.91, 43.60, 28.64. HRMS (ESI+): C25H31N4O5 + [MH]+ calcd: 467.2289, found: 467.2287. (PNG) [file pone.0133805.s010.png]
